# Lysophosphatidylethanolamine (LysoPE) in health and disease: a narrative review of its pathophysiological mechanisms and translational potential as a biomarker

**DOI:** 10.3389/fcell.2026.1852277

**Published:** 2026-05-20

**Authors:** Ke-Mu Wang, Xue-Ying Shang, Xiao-Guang Shi

**Affiliations:** 1 Department of Endocrinology and Metabolism, Shengjing Hospital of China Medical University, Shenyang, Liaoning, China; 2 Department of Endocrinology, The Fourth Affiliated Hospital of China Medical University, Shenyang, China

**Keywords:** biomarker, lysophosphatidylethanolamine, metabolic diseases, neurodegenerative diseases, signaling pathways, tumor

## Abstract

Lysophosphatidylethanolamine (LysoPE) is a key class of lysophospholipid molecules that play important roles in regulating cell membrane structure, signal transduction, and metabolic regulation. This review consolidates current knowledge on LysoPE’s molecular mechanisms, including its biosynthesis, membrane dynamics, and signaling via GPCRs, MAPK, and PI3K/AKT pathways. We critically analyze its context-dependent roles across major disease categories. In metabolic diseases, abnormal LysoPE levels are associated with lipid accumulation and insulin resistance. In the nervous system, LysoPE contributes to neuroinflammation while also exerting neurotrophic and protective effects through MAPK signaling. In tumor progression, LysoPE exhibits tissue-specific pro-migratory or inhibitory effects. LysoPE also shows pathological relevance in cardiovascular diseases, infections, and immune abnormalities. Based on disease-specific expression changes, LysoPE has shown promise as a candidate diagnostic biomarker in preliminary studies, with demonstrated value in treatment efficacy evaluation and prognosis prediction. However, large-scale prospective validation is required before clinical application. Future research should integrate multi-omics technologies and clinical validation to further elucidate its molecular mechanisms and promote its application in precision medicine.

## 1 Introduction

Lysophosphatidylethanolamine (LysoPE) is a lysophospholipid generated through partial hydrolysis of phosphatidylethanolamine (PE) by enzymes such as phospholipase A_2_ (PLA_2_) and serves as an essential component of cell membranes, which attracts increasing attention in recent years. Beyond its structural role in cellular membranes, LysoPE is also involved in regulating membrane fluidity, signal transduction, and intercellular communication, contributing to vital physiological functions. Its production involves multiple forms of PLA_2_, including platelet-activating factor acetylhydrolase (PAF-AH) in plasma and sPLA_2_, which play key roles in LysoPE generation and exhibit high activity particularly during the cleavage of oxidized phospholipids (Jokesch et al., 2025). Furthermore, the concentration and molecular species of LysoPE both intra- and extracellularly significantly influence its biological activity. For example, LysoPE species with saturated (e.g., 16:0, 18:0) or polyunsaturated (e.g., 20:4, 22:6) fatty acid chains exhibit distinct biological activities. Advances in analytical techniques, especially the development of liquid chromatography-tandem mass spectrometry (LC-MS/MS), now allow more precise quantification and identification of LysoPE molecular species in serum and tissues, providing a robust technical foundation for deeper investigation into the physiological and pathological functions of LysoPE (Yamamoto et al., 2021).

The role of LysoPE in the regulation of cellular metabolism is gradually being revealed, particularly in studies on its function in lipid metabolism within hepatocytes. Research indicates that LysoPE can promote the formation of lipid droplets, inhibit fatty acid biosynthesis and triacylglycerol hydrolysis, thereby leading to lipid accumulation. This mechanism suggests that LysoPE may be involved in the occurrence and development of metabolic diseases, such as fatty liver disease. Supplementation of human hepatocyte lines with LysoPE 18:2 demonstrated its ability to modulate the expression of lipid metabolism-related genes. Specifically, it downregulated adipose triglyceride lipase (ATGL) as well as key fatty acid synthesis regulators, including sterol regulatory element-binding protein 1 (SREBP1) and stearoyl-CoA desaturase 1 (SCD1). These findings further elucidate the regulatory role of LysoPE in maintaining lipid metabolic balance (Yamamoto et al., 2022). In addition, alterations in the circulating levels of LysoPE and its metabolites are associated with various disease states. For instance, significant reductions in multiple LysoPE molecular species have been observed in the serum of patients with non-alcoholic fatty liver disease (NAFLD), suggesting that LysoPE may serve as a potential biomarker for the disease (Yamamoto et al., 2021).

In addition, growing attention has been paid to the role of LysoPE in blood–brain barrier (BBB) transport and the nervous system. Studies have shown that docosahexaenoic acid (DHA)-containing LysoPE (LysoPE-DHA) can cross an *in vitro* human BBB model and be taken up by brain cells more efficiently than unesterified DHA or LysoPC-DHA. This suggests that LysoPE may serve as an effective carrier for essential fatty acids such as DHA, facilitating their delivery to brain tissue and thereby influencing neurological health and disease (Hachem et al., 2024). These findings provide a novel perspective for future applications of LysoPE as a delivery vehicle or therapeutic target.

In this narrative review, we aim to: (i) systematically consolidate current knowledge on the biosynthesis, transport, and signaling mechanisms of LysoPE; (ii) critically analyze its context-dependent pathophysiological roles across major disease categories; (iii) evaluate its emerging potential as a diagnostic and prognostic biomarker; and (iv) identify knowledge gaps and propose future research directions for clinical translation.

## 2 Structure and biosynthesis/metabolic pathways of LysoPE

LysoPE is a class of lysophospholipids composed of a glycerol backbone, a single fatty acid chain, and a PE head group. The glycerol backbone of LysoPE carries only one fatty acid chain, while the other position is typically occupied by a hydroxyl group, imparting relatively high hydrophilicity and low hydrophobicity. This structural characteristic endows LysoPE with distinct properties in terms of membrane localization and dynamic behavior within cellular membranes. LysoPE molecular species vary depending on the fatty acid chain attached, which can be saturated (e.g., 16:0, 18:0) or polyunsaturated (e.g., 20:4, 22:6 docosahexaenoic acid, DHA), with different species exhibiting distinct biological activities (Yamamoto et al., 2021). The PE head group contains amine and phosphate moieties, which confer hydrophilicity and enable LysoPE to engage in specific interactions with membrane proteins, receptors, or other molecules (Đukanović et al., 2021).

In human serum, LysoPE concentrations range from approximately 10–50 μM (while in mice, the range is 4–6 μM), accounting for about 1% of total serum phospholipids (Quehenberger et al., 2010). Albumin in the blood serves as the carrier for LysoPE (Tan et al., 2020).

The synthesis of LysoPE primarily relies on the hydrolysis of PE by PLA_2_, which specifically cleaves the ester bond at the sn-2 position of the glycerol backbone, generating LysoPE and a free fatty acid (Tan et al., 2020). This sn-2 specificity has been confirmed in human plasma, where only sn-2 lysophospholipids accumulate after incubation with oxidized PE (Jokesch et al., 2025). Alternatively, LysoPE can also be generated through the action of phospholipase A_1_ (PLA_1_) at the sn-1 position. In addition to these hydrolytic pathways, LysoPE can be formed through the acylation of glycerophosphoethanolamine, an intermediate of the Kennedy pathway for *de novo* PE biosynthesis, or via the deacylation of PE through the reverse reaction of lysophospholipid acyltransferases (Farine et al., 2015). Studies have shown that human plasma contains multiple PLA_2_ subtypes, with platelet-activating factor acetylhydrolase (PAF-AH, also known as Lp-PLA_2_) and secretory phospholipase A_2_ (sPLA_2_) being predominant. These enzymes also exhibit strong hydrolytic activity toward oxidized phospholipids (OxPLs) and play a leading role in the generation of LysoPE. Additionally, intracellular enzymes such as peroxiredoxin-6 are involved in the metabolism of oxidized phospholipid substrates, indicating that the production of LysoPE is regulated by multiple enzymatic pathways (Jokesch et al., 2025).

**FIGURE 1:**
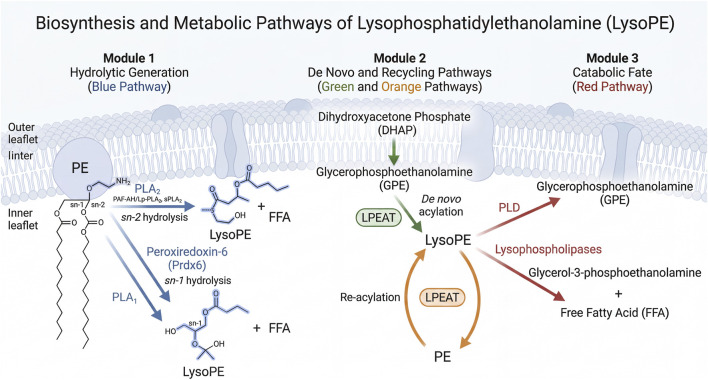
Biosynthesis and Metabolic Pathways of Lysophosphatidylethanolamine (LysoPE). The synthesis and fate of LysoPE are organized into three interconnected modules. Module 1 (Hydrolytic Generation, blue): Phosphatidylethanolamine (PE) embedded in the inner leaflet of the plasma membrane is hydrolyzed by phospholipase A_2_ (PLA_2_) at the sn-2 position, generating LysoPE and a free fatty acid. Major plasma PLA_2_ subtypes include PAF-AH/Lp-PLA_2_ and sPLA_2_, while peroxiredoxin-6 (Prdx6) contributes intracellularly. Alternatively, phospholipase A_1_ (PLA_1_) cleaves the sn-1 position. Module 2 (*De Novo* and Recycling, green and orange): LysoPE can be synthesized *de novo* through the acylation of glycerophosphoethanolamine (GPE), an intermediate derived from dihydroxyacetone phosphate (DHAP) in the Kennedy pathway. This reaction is catalyzed by lysophospholipid acyltransferases (LPEATs). In the recycling pathway, LysoPE is re-esterified to PE by LPEATs, maintaining membrane phospholipid homeostasis. Module 3 (Catabolic Fate, red): LysoPE is degraded by phospholipase D (PLD) to GPE, or by lysophospholipases to glycerol-3-phosphoethanolamine and free fatty acid.

The metabolism of LysoPE extends beyond its synthesis to include complex degradation and resynthesis processes (Figure 1). LysoPE can be re-esterified to form PE, thereby participating in the recycling of phospholipids (Takagi et al., 2022). This process is critical for maintaining the integrity and function of cell membranes. Additionally, phospholipase D (PLD) is involved in the conversion of LysoPE by catalyzing the hydrolysis of phospholipid molecules, thereby regulating the levels of LysoPE and its related metabolites (Aoki et al., 2008).

## 3 Transport and cellular distribution of LysoPE

Since LysoPE is also a zwitterionic lipid, its transport across the plasma membrane may be mediated by protein transporters. PE is primarily located in the inner leaflet of the plasma membrane and is hydrolyzed to produce LysoPE. The release of LysoPE from the inner leaflet to the outer leaflet likely requires the involvement of protein transporters. Similarly, the plasma membrane may also express LysoPE transporters for the uptake of circulating LysoPE. In addition, Mfsd2a (Major Facilitator Superfamily Domain Containing 2A) is also capable of binding LysoPE. Mfsd2a is a key transporter for maintaining the function of the blood-brain barrier and cerebral homeostasis. It specifically transports omega-3 fatty acids (such as DHA) in the form of lysophosphatidylcholine into the brain, serving as the primary pathway for the brain’s uptake of these essential nutrients (Nguyen et al., 2014). Furthermore, Mfsd2a inhibits vesicular transcytosis in endothelial cells of the blood-brain barrier through its unique transport mechanism, thereby structurally maintaining the low permeability and integrity of the blood-brain barrier (Ben-Zvi et al., 2014; Andreone et al., 2017). Therefore, Mfsd2a is not only essential for brain development during the embryonic stage, but its dysfunction is also associated with various neurological disorders (Ben-Zvi et al., 2014; Nguyen et al., 2014). The brain is rich in various PE species containing essential fatty acids (such as DHA). Whether plasma LysoPE is taken up via Mfsd2a for PE synthesis in neuronal cells requires further investigation; how LysoPE is released into the bloodstream also needs further exploration (Tan et al., 2020). The asymmetric distribution and regulated transport of LysoPE across membrane leaflets not only maintain phospholipid homeostasis but also influence the biophysical properties of cellular membranes. The following section discusses how dynamic changes in LysoPE levels contribute to membrane remodeling under physiological and pathological conditions.

## 4 Regulation of cell membrane dynamics by LysoPE

The differential distribution of LysoPE in various regions of the cell membrane is closely related to dynamic changes in membrane structure, particularly under cellular stress conditions. Changes in LysoPE content correspond to adjustments in membrane architecture, aiding cells in adapting to environmental variations (Miranda et al., 2019; Krupska et al., 2021). Illustrating this concept, a study using a gerbil model of ischemia–reperfusion (IR) found that under normal conditions (control group), certain LysoPE species (such as 18:1 and 22:6) in the hippocampal CA1 region (the vulnerable area) were significantly higher than in the CA2–4/DG region (the resistant area), indicating intrinsic heterogeneity in lipid composition within the hippocampus. Following ischemia–reperfusion, changes in LysoPE diverged between the two regions: the difference in LysoPE 18:1 between the two areas further widened after ischemia (in the IR group, CA1 was 107% higher than CA2–4/DG), while LysoPE 20:4 significantly decreased in the CA1 region after IR (81% lower compared to its own control). When comparing the IR groups, the content in CA1 was also substantially lower than in CA2–4/DG. This distinctive and uneven lipid profile may reflect heterogeneity in the membrane lipid composition between the two regions. Such heterogeneity could lead to differences in membrane permeability, stability, and electrical properties, ultimately influencing neuronal excitability (Krupska et al., 2021). Beyond the central nervous system, LysoPE also exhibits a role in regulating membrane permeability during pathogenic bacterial infection. For example, the human pathogen *Campylobacter* jejuni utilizes its membrane-enriched LysoPE to disrupt and penetrate host cell membranes, highlighting the critical regulatory function of LysoPE in both physiological and pathological states of cell membranes (Cao et al., 2022).

## 5 Role of LysoPE in cellular signal transduction

**FIGURE 2:**
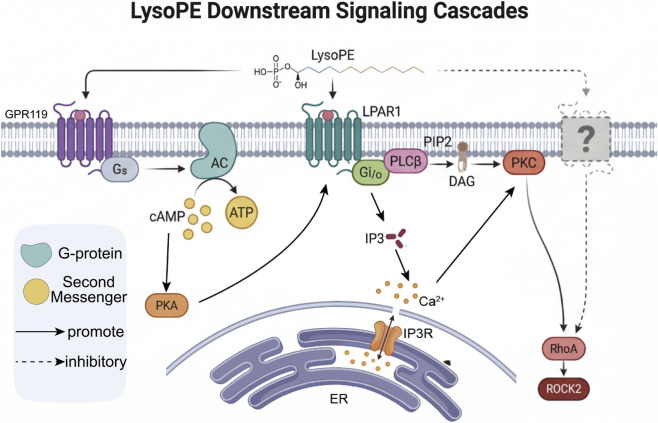
LysoPE Downstream Signaling Cascades. Schematic overview of the major signaling pathways activated by LysoPE. LysoPE signals through multiple GPCR-dependent mechanisms, including GPR119-mediated cAMP/PKA activation, Gαi/o-mediated calcium mobilization via Gβγ-PLC-IP_3_, and Gαq/11-mediated PLCβ activation leading to calcium release and PKC activation. In addition, LysoPE activates receptor tyrosine kinase-coupled pathways including the MAPK/ERK cascade (EGFR→MEK1/2→ERK1/2) and the PI3K/AKT pathway, which collectively regulate cell survival, proliferation, differentiation, and apoptosis. The RhoA/ROCK2 axis mediates cytoskeletal reorganization downstream of LysoPE stimulation. Solid arrows indicate experimentally validated pathways; dashed arrows indicate proposed or indirect connections. See Section 5 for detailed mechanistic descriptions (Soga et al., 2005; Nishina et al., 2006; Overton et al., 2008; Lee et al., 2017; Tan et al., 2020).

LysoPE participates in intercellular signaling and can activate signaling enzymes (Lee et al., 2017), potentially functioning through G-protein-coupled receptors (GPCRs) (Soga et al., 2005). The downstream signaling cascades of LysoPE are summarized in Figure 2.

Studies indicate that LysoPE can induce calcium signaling via pertussis toxin-sensitive G i/o proteins and edelfosine-sensitive phospholipase C. In the Gαi/o-dependent pathway, LysoPE binding to its cognate GPCR leads to the dissociation of Gαi/o and Gβγ subunits; the Gβγ dimer subsequently activates phospholipase C (PLC), which hydrolyzes phosphatidylinositol 4,5-bisphosphate (PIP_2_) to generate inositol 1,4,5-trisphosphate (IP_3_) and diacylglycerol (DAG). IP_3_ binds to IP_3_ receptors on the endoplasmic reticulum, triggering Ca^2+^ release into the cytoplasm. Alternatively, LysoPE may activate Gαq/11-coupled receptors, leading to PLCβ activation and PIP_2_ hydrolysis through a Gαq/11-dependent mechanism, similarly resulting in IP_3_-mediated Ca^2+^ release and DAG-mediated protein kinase C (PKC) activation. Moreover, in MDA-MB-231 and PC-12 cells, LysoPE-induced calcium influx depends on the lysophosphatidic acid receptor 1 (LPAR1) (Tan et al., 2020). However, its receptor identity remains controversial, and the specific receptor for LysoPE in SK-OV3 cells has not yet been clarified (Lee et al., 2017). On the other hand, in rat hepatoma RH7777 cells stably expressing GPR119, LysoPE can bind to GPR119, which couples to Gαs, leading to adenylyl cyclase activation and intracellular cAMP accumulation (Soga et al., 2005; Overton et al., 2008).

In addition to GPCR-mediated signaling, LysoPE can also influence apoptotic and survival signaling by modulating key intracellular pathways. Through the MAPK/ERK cascade, LysoPE activates the epidermal growth factor receptor (EGFR), which in turn triggers the phosphorylation of MEK1/2 and ERK1/2, promoting cell survival, neuronal differentiation, and neurite outgrowth. LysoPE also activates the PI3K/AKT pathway, which regulates downstream effectors involved in cell survival, metabolism, and inhibition of apoptosis. Importantly, LysoPE has been shown to modulate the RhoA/ROCK2 signaling axis, which governs cytoskeletal reorganization and influences cell migration and morphological changes. The interplay between these pathways enables LysoPE to exert pleiotropic effects on fundamental cellular processes, with the specific outcome depending on cell type, receptor expression profile, and cellular context (Nishina et al., 2006; Zhang et al., 2024).

## 6 Association of LysoPE with infectious, immune, and inflammatory diseases

LysoPE exhibits distinct metabolic changes and regulatory functions across different disease contexts, with disease- and environment-specific effects.

In patients with rheumatoid arthritis (RA), fecal metabolites show a marked increase in LysoPE levels (Yu et al., 2021). In a collagen-induced arthritis (CIA) mouse model, reduced LysoPE 14:0 was associated with increased ferroptosis, while GSK484 intervention partially restored LysoPE 14:0 levels, thereby improving microbial homeostasis and alleviating joint inflammation (Zhu et al., 2025).

In a rat model of acute diarrhea, LysoPE levels positively correlated with pro-inflammatory cytokines (IL-6, IL-1β, TNF-α, IFN-γ) and pathogen abundance, suggesting that LysoPE may play a promoting role in intestinal inflammation and mucosal injury (Madjirebaye et al., 2024).

Other studies have demonstrated that transferring respiratory microbiota from mice that survived H9N2 influenza to antibiotic-treated mice enhanced resistance to infection in the recipients. Metabolomic analysis revealed elevated LysoPE 16:0 in mildly infected mice. Further *in vivo* and *in vitro* experiments confirmed that LysoPE 16:0 suppresses inducible nitric oxide synthase (iNOS) and cyclooxygenase-2 (COX-2) expression, thereby enhancing anti-influenza defense (Yan et al., 2024).

Having discussed the roles of LysoPE in infectious and inflammatory conditions, we now turn to its pathophysiological functions in metabolic diseases, where lipid dysregulation plays a central role.

## 7 Pathophysiological role of LysoPE in metabolic diseases

### 7.1 LysoPE and NAFLD/NASH as well as lipid metabolic disorders

**FIGURE 3:**
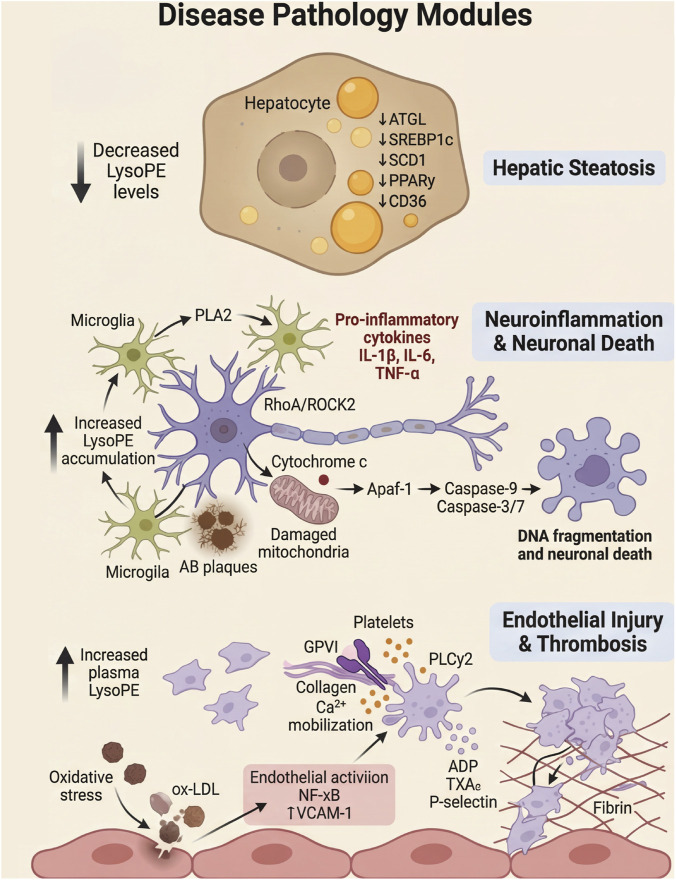
LysoPE in Disease Pathway Modules. This figure integrates key findings on LysoPE dysregulation across metabolic, neurological, and oncological diseases, highlighting common pathways (e.g., SREBP1, MAPK, PI3K/AKT) and tissue-specific effects.

A small-scale clinical study compared serum LysoPE levels between healthy subjects (n = 8) and NAFLD patients [patients with simple steatosis (SS, n = 9) and those with non-alcoholic steatohepatitis (NASH, n = 27)]. The results showed that healthy subjects had the highest LysoPE levels at 18.030 ± 3.832 nmol/mL, while SS patients had 4.867 ± 1.852 nmol/mL, and NASH patients had 5.497 ± 2.495 nmol/mL (Yamamoto et al., 2021). With the exception of LysoPE 18:0, all other LysoPE species showed significant reductions, particularly LysoPE 18:2, 20:4, 20:5, and 22:6. Moreover, there was no significant difference between the SS group and the NASH group (Yamamoto et al., 2021). In another study conducted in Nanjing, China, similar conclusions were reached. This research compared the serum metabolomics of healthy subjects (n = 50) and patients with non-alcoholic fatty liver disease (NAFLD) (n = 50). The results showed decreased levels of LysoPE (18:0/0:0) and LysoPE (0:0/20:2), but an increase in LysoPE (0:0/22:1) (Yang et al., 2021) (Figure 3).

Supplementation of human hepatocyte lines with LysoPE 18:2 revealed its ability to modulate the expression of lipid metabolism-related genes. It downregulated adipose triglyceride lipase (ATGL) and fatty acid synthesis regulators SREBP1 and SCD1, as well as decreased fatty acid transport-related genes PPARγ and CD36, demonstrating the regulatory role of LysoPE in lipid metabolic balance (Yamamoto et al., 2022).

Animal studies have yielded similar conclusions. In the livers of NASH model mice, the levels of C18:2-LysoPE and C20:5-LysoPE were significantly lower than in the control group. With the exception of C20:4, other LysoPE species also showed a decreasing trend in NASH, which aligns with the lipidomic changes observed in human NASH patients (Furukawa et al., 2016). In high-fat diet-induced obese mice, hepatic LysoPE (0:0/18:0) was identified as a potential lipid biomarker (Xie et al., 2023). Treatment with heat-processed *G. pentaphyllum* (HGyp) downregulated genes and proteins related to lipogenesis (SREBP1, ACC1, SCD1), upregulated genes and proteins involved in lipid oxidation (PPARα, CPTA1), and consequently improved lipid metabolism disorders and hepatic steatosis (Xie et al., 2023). Similarly, in a high-fat diet-induced obese rat model, treatment with black chokeberry polyphenols (BCPs) effectively increased serum LysoPE levels (such as LysoPE 24:0 and 24:1). This improvement was achieved by markedly modulating the mRNA and protein expression levels of genes involved in glycerophospholipid metabolism signaling pathways (e.g., PPARα, CPT1α, EPT1, LCAT), thereby ameliorating lipid metabolism disorders and hepatic steatosis (Zhu et al., 2022).

In adults with growth hormone deficiency (AGHD) and concurrent intracranial germ cell tumors (iGCTs), LysoPE-O showed a negative correlation with metabolic indicators such as fasting insulin, Homeostatic Model Assessment for Insulin Resistance (HOMA-IR), and triglycerides, suggesting its potential as a biomarker for metabolic health in AGHD patients (Yang H. et al., 2025). In subretinal drusenoid deposits/reticular pseudodrusen (SDD), a high-risk phenotype of age-related macular degeneration, LysoPE is a key component of lipid metabolism disturbance, particularly LysoPE (18:0) (Anderson et al., 2023).

Furthermore, exposure to environmental pollutants such as PFBS during pregnancy also affects offspring lipid metabolism. Alterations in the concentration of LysoPE 18:1 have been identified as one of the biomarkers of lipid metabolism disruption (Meng et al., 2023). This evidence indicates that LysoPE not only serves as a marker of lipid metabolism but its abnormal alterations may also be involved in the pathological processes of diseases associated with lipid metabolism disorders (Figure 3). A comprehensive summary of LysoPE dysregulation across major diseases is provided in Table 1.

**TABLE 1:** Summary of LysoPE dysregulation across diseases.

Disease/Condition	LysoPE change	Key molecular pathways	Clinical outcome/Association	References
NAFLD/NASH	↓ (multiple species: 18:2, 20:4, 22:6)	SREBP1, SCD1, ATGL, PPARα	Hepatic steatosis, biomarker potential	Yamamoto et al., 2021; Yamamoto et al., 2022
Type 2 diabetes/Insulin resistance	↓/↑ (context-dependent)	Insulin signaling, HOMA-IR, chronic inflammation	Insulin resistance, β-cell dysfunction	Meikle and Summers, 2017; Pang et al., 2024
Cardiovascular disease (CAD)	↑ (platelet-associated species)	GPVI, PLCγ2, platelet aggregation	Thrombosis, adverse cardiovascular events	Harm et al. (2023)
Alzheimer’s disease	↑ (plaque-associated accumulation)	PLA_2_, neuroinflammation, MAPK	Aβ plaque accumulation, cognitive decline	Kaya et al., 2020; Hong et al., 2016
Rheumatoid arthritis	↑ (fecal metabolites)	Ferroptosis (LysoPE 14:0↓ with GSK484 intervention)	Joint inflammation, microbial homeostasis	Yu et al., 2021; Zhu et al., 2025
Colorectal cancer	↓ (tissue and serum, e.g., 17:1, 22:6)	Lipid metabolic reprogramming	Diagnostic biomarker, location-specific differences	Kang et al., 2023; Amir Hashim et al., 2021
Breast cancer	↑ LysoPE (18:1), (18:2) in TNBC non-responders to NACT	Lipid remodeling under metabolic stress	Treatment response prediction	Díaz et al. (2022)
Non-small cell lung cancer	Altered serum LysoPE	Disease-related metabolic changes	Diagnostic potential	Zhao et al. (2021)
Cervical cancer	↑ LysoPE (18:1) after cisplatin-based chemoradiotherapy	Treatment-induced metabolic changes	Treatment monitoring	Choi et al. (2024)

### 7.2 Role of LysoPE in diabetes

Firstly, LysoPE participates in glucose metabolism abnormalities by modulating pancreatic β-cell function. In the db/db diabetic mouse model, alterations in LysoPE are associated with disturbances in fatty acid and phospholipid metabolism. These metabolic pathway dysregulations contribute to increased oxidative stress and apoptosis in β-cells, thereby impairing the secretory function of pancreatic islet β-cells (Jiang et al., 2022).

Secondly, LysoPE influences the insulin signaling pathway and participates in the development of insulin resistance. Various phospholipid molecules in plasma, including LysoPE, show correlations with insulin resistance indicators such as HOMA-IR (Meikle and Summers, 2017). Specifically, in diabetic patients and insulin-resistant individuals, alterations in LysoPE levels reflect abnormalities in insulin signaling pathways. These changes may interfere with the function of insulin receptors and downstream signaling molecules by affecting phospholipid composition and membrane protein activity in cellular membranes (Lu et al., 2023; Pang et al., 2024). Furthermore, LysoPE-associated lipid metabolism dysregulation also affects fatty acid metabolism in adipose tissue and liver, promoting lipotoxicity and further aggravating insulin resistance. Simultaneously, elevated LysoPE levels in diabetic patients correspond with the chronic inflammatory state associated with insulin resistance (Pang et al., 2024).

### 7.3 Association of LysoPE with cardiovascular diseases

Multiple studies using metabolomic and lipidomic approaches have revealed that abnormal LysoPE levels under cardiovascular pathological conditions may serve as disease biomarkers and potential pathological mediators. In patients with borderline hypercholesterolemia, elevated levels of oxidized low-density lipoprotein (ox-LDL) are significantly associated with increased plasma LysoPE content. This alteration is closely linked to coagulation dysfunction and vascular endothelial injury, suggesting that LysoPE is involved in the formation of a pro-coagulant state (Kim et al., 2020). As a key component of the HDL lipidome, aberrant expression of LysoPE may impair the antioxidant and anti-inflammatory functions of HDL, thereby affecting cardiovascular health (Shimura et al., 2025). A retrospective study examining the relationship between platelet lipidomes and adverse cardiovascular events in patients with coronary artery disease found that 13 LysoPE species were significantly upregulated in patients who experienced cardiovascular events. Moreover, high levels of LysoPE were strongly associated with enhanced collagen-induced platelet aggregation. Cox proportional hazards models revealed that 14 LysoPE species [including, for example, LysoPE (20:1/0:0), LysoPE (0:0/18:1), and LysoPE (0:0/20:3)] were independently associated with increased cardiovascular risk, suggesting a potential role of LysoPE in platelet-mediated thrombosis and inflammation (Harm et al., 2023).

In a rat model of coronary heart disease, LysoPE (18:2/0:0) significantly decreased, while LysoPE (20:4/0:0) significantly increased. Following treatment with salvianolic acid B (Sal B), the levels of both LysoPE species tended to normalize. These alterations reflect lipid metabolism disturbances in the coronary heart disease model, particularly phospholipid metabolic imbalance (Li et al., 2020).

Furthermore, alterations in gut microbiota among HIV-infected individuals are collectively associated with plasma LysoPE and related lipid metabolic abnormalities, thereby promoting carotid plaque formation. This suggests a cross-talking role of LysoPE in immune regulation and atherosclerosis (Wang et al., 2022).

Pseudo-targeted metabolomic analysis revealed significant alterations in LysoPE in a rat model of cardiac hypertrophy (CH), involving key pathways such as tyrosine metabolism and arachidonic acid metabolism, reflecting its potential role in myocardial structural and functional abnormalities (Liu et al., 2024). In a mouse model of myocardial ischemia-reperfusion injury, a compound preparation of Salvia miltiorrhiza exerted anti-inflammatory and cardioprotective effects by modulating lipid metabolites such as LysoPE, further verifying the importance of LysoPE in the process of myocardial injury repair (Zhang et al., 2024).

Beyond metabolic regulation, LysoPE also plays significant roles in the nervous system, where it exerts both neuroinflammatory and neurotrophic effects.

## 8 Role of LysoPE in neurological disorders

### 8.1 LysoPE and neuroinflammation

In patients with vascular cognitive impairment, serum LysoPE levels are significantly elevated and are associated with increased activity of PLA_2_(Liu et al., 2023). Increased PLA_2_ neuronal activity converts PC into LPC, thereby stimulating glial cells and inducing neuroinflammation (Sundaram et al., 2012). Studies have found that in the brains of 5xFAD mice, a model of Alzheimer’s disease (AD), LysoPE accumulates in β-amyloid plaques (Aβ) (Kaya et al., 2020) and may activate PLA_2_, thereby triggering neuroinflammation (Hong et al., 2016). Similarly, significant changes in LysoPE were observed in the brains of APP/PS1 mice (Liu et al., 2022). This suggests that alterations in LysoPE metabolism are closely associated with neuroinflammation, neurodegeneration, and cognitive dysfunction (Figure 3).

Furthermore, in an animal model of cerebral ischemia/reperfusion injury, treatment with a combination of Dragon’s Blood (Dracaena Blood Resin) and borneol significantly elevated the levels of multiple LysoPE species, such as LysoPE (18:1), LysoPE (18:0), LysoPE (20:1), and LysoPE (20:0). Moreover, LysoPE (22:4) showed the most significant correlation with the IL-17 signaling pathway (Ren et al., 2024). When combined with extracts of Eucommia ulmoides (EUE), LysoPE (0:0/20:4) significantly suppressed neuronal apoptosis and inflammatory responses, alleviating cerebral ischemic injury, indicating the potential of LysoPE in neuroprotection and anti-inflammation (Qi et al., 2025).

### 8.2 Impact of LysoPE on neuronal survival and apoptosis

Firstly, LysoPE plays a key role in regulating apoptotic signaling pathways. In a cerebral ischemia-reperfusion injury model, the traditional Chinese medicine compound Buyang Huanwu Decoction (BYHWD) was found to significantly reduce LysoPE levels by modulating the phenylalanine metabolism pathway, thereby inhibiting the activation of the RhoA/Rock2 signaling pathway and reducing mitochondria-mediated neuronal apoptosis (Li et al., 2025). Furthermore, modulation of LysoPE metabolism may also reduce the expression of pro-apoptotic proteins and enhance the activity of anti-apoptotic factors, thereby overall promoting neuronal survival (Li et al., 2025).

Secondly, LysoPE also plays an important role in the repair mechanisms following neural injury. Studies have shown that after exposure to the insecticide bifenthrin, levels of LysoPE and other lysophospholipids in the brain significantly decreased, accompanied by alterations in the expression of genes associated with neuroinflammation and apoptosis. This suggests that changes in LysoPE levels may affect neuronal repair capacity and functional recovery (Magnuson et al., 2020).

Furthermore, in an *in vitro* model of Alzheimer’s disease using the mouse neuroblastoma cell line (N2A), the content of LysoPE and its associated lipidomic profile changed within the mitochondria-associated endoplasmic reticulum membranes (MAMs) (Fernandes et al., 2024). MAMs are important subcellular structures regulating lipid metabolism and apoptosis. The reduction of LysoPE may lead to lipid metabolic imbalance, exacerbating mitochondrial dysfunction and endoplasmic reticulum stress, thereby promoting neuronal apoptosis and disease progression.

### 8.3 Potential role of LysoPE in neurotrophism and regeneration

LysoPE not only exerts neurotrophic and neuroprotective effects but also promotes neuronal differentiation (Nishina et al., 2006). In PC12 cells, LysoPE activates MAPK through the EGFR→MEK1/2→ERK1/2 signaling pathway—not via TrkA or G-protein-coupled receptors—exerting neurotrophic and neuroprotective effects. Meanwhile, LysoPE reduces cell condensation and DNA laddering, thereby inhibiting serum deprivation-induced apoptosis in PC12 cells (Nishina et al., 2006). In addition, LysoPE induces morphological changes such as neurite outgrowth in PC12 cells and upregulates the expression of neurofilament-M (NF-M), demonstrating a pro-differentiation effect similar to that of nerve growth factor (NGF) (Nishina et al., 2006).

The dual role of LysoPE in promoting or suppressing cellular processes extends to oncological diseases, where it exhibits tissue-specific effects on tumor progression.

## 9 Role of LysoPE in oncological diseases

### 9.1 Impact of LysoPE on tumor cell migration and invasion

LysoPE significantly enhances chemotactic migration and promotes cellular invasion in SK-OV3 ovarian cancer cells (Park et al., 2007). However, in MDA-MB-231 breast cancer cells, although LysoPE can induce calcium influx, it does not exhibit a significant promoting effect on migration or invasion (Park et al., 2013). This may be because LysoPE has relatively weak signaling strength compared to lysophosphatidic acid (LPA) and does not activate additional migration-related pathways that LPA engages (Figure 3).

### 9.2 Potential of LysoPE as a diagnostic and therapeutic target in tumors

Accumulating evidence suggests that altered LysoPE levels are associated with various cancer types, although their utility as definitive biomarkers requires further validation. In a study involving patients with stage I-III colorectal cancer (CRC) who had not received prior chemotherapy, metabolomic analysis revealed that LysoPE (17:1/0:0) was significantly downregulated in tumor tissues compared to adjacent non-tumor tissues, and LysoPE levels in the right-sided colon were notably higher than those in the left-sided colon and rectum (P < 0.05), suggesting its possible involvement in lipid metabolic reprogramming as a location-specific metabolic feature rather than a universal diagnostic marker (Kang et al., 2023). Similarly, in a cohort of Malaysian CRC patients (predominantly stage II-III), serum LysoPE (22:6) was significantly downregulated compared to healthy controls, and ROC curve analysis showed that it had moderate sensitivity and specificity as a candidate diagnostic biomarker (Amir Hashim et al., 2021).

In patients with non-small cell lung cancer (NSCLC), serum LysoPE levels showed disease-related alterations, although the specific molecular species varied across studies (Zhao et al., 2021). In a cohort of locally advanced cervical cancer patients receiving concurrent cisplatin-based chemoradiotherapy, serum LysoPE (18:1) levels were significantly elevated after treatment, although the functional significance of this increase remains to be determined (Choi et al., 2024). In a prospective study of breast cancer patients undergoing neoadjuvant chemotherapy (NACT), plasma LysoPE dynamics were analyzed at multiple time points. Specifically, in triple-negative non-responder (TN NR) patients (stage II-III), LysoPE (18:1) and (18:2) concentrations increased at the first time point (t1) during NACT, whereas no such changes were observed in responders or other subtypes. This seemingly paradoxical finding—increased LysoPE in non-responders—highlights the context- and subtype-specific nature of LysoPE dynamics during cancer treatment. Phosphatidylethanolamine (PE) is a major membrane phospholipid, and its lysophospholipid derivative LysoPE is generated via PLA_2_-mediated hydrolysis. The elevation of these PE-derived lysophospholipids in non-responders may reflect an increased cellular demand for PE under metabolic stress in aggressive breast cancer subtypes, as rapidly proliferating tumor cells require enhanced membrane biogenesis and lipid remodeling (Díaz et al., 2022). Collectively, these studies indicate that LysoPE and its related metabolites hold potential as biomarkers for tumor diagnosis and therapeutic monitoring.

Beyond colorectal, breast, lung, and cervical cancers, lipidomics profiling has revealed altered LysoPE levels in other malignancies. In platinum-resistant gastric cancer patients, serum LysoPE (18:0) and LysoPE (18:1) were significantly decreased compared to healthy controls, correlating with tumor stage (Chen et al., 2024). In patients with hepatocellular carcinoma (HCC), tissue LysoPE species, particularly those containing polyunsaturated fatty acids, showed downregulation compared to adjacent non-tumor tissues, and lower levels were associated with poor prognosis (Ursu et al., 2025). These findings suggest that LysoPE dysregulation may be a common feature of cancer-associated lipid metabolic reprogramming, although the direction and magnitude of changes appear to be cancer-type specific.

It should be noted, however, that the specific receptor(s) mediating LysoPE’s effects in cancer cells remain unidentified, and the expression patterns and underlying mechanisms of LysoPE dysregulation require further investigation across diverse cancer types and larger patient cohorts.

## 10 Prospects for clinical application and future research directions of LysoPE

### 10.1 Potential application of LysoPE in disease diagnosis

First, multiple studies support the use of LysoPE as a blood-based biomarker. Regarding non-alcoholic fatty liver disease (NAFLD), research has found that total serum LysoPE and specific molecular species are significantly lower in patients compared with healthy controls, with little difference between those with simple steatosis and non-alcoholic steatohepatitis. This suggests that decreased LysoPE levels are associated with the presence of NAFLD, offering a novel hematological indicator for the diagnosis of the disease (Yamamoto et al., 2021). Furthermore, in traumatic coagulopathy (TIC), two molecular species—LysoPE (20:4) and LysoPE (0:0/18:2)—have been identified as biomarkers with high diagnostic efficacy, achieving areas under the curve (AUC) of 0.933 and 0.916, respectively, outperforming traditional coagulation assays (Xu et al., 2025). Similarly, in metabolomic studies of spinal tuberculosis (Wang et al., 2024) and sepsis (Peng et al., 2023), LysoPE molecules have also been confirmed to possess significant diagnostic value, indicating their potential application in infectious diseases.

Secondly, the application of LysoPE combined with multi-omics technologies significantly improves the accuracy and sensitivity of disease diagnosis. Using liquid chromatography-tandem mass spectrometry (LC-MS/MS), high-sensitivity and high-throughput detection of serum LysoPE molecules enables simultaneous quantification of multiple molecular species, greatly enriching the molecular profile of disease biomarkers (Yamamoto et al., 2021). Integrating metabolomics with clinical indicators in combined modeling provides a more comprehensive reflection of the pathophysiological state of diseases. For example, metabolomic analysis of patients with systemic lupus erythematosus (SLE) has shown that alterations in lipid molecules such as LysoPE correlate with disease activity, and incorporating multi-omics data can help improve the accuracy of early diagnosis (Zhang et al., 2021). In studies predicting the efficacy of neoadjuvant chemotherapy (NACT) for breast cancer, dynamic changes in LysoPE have been used to assist in identifying treatment responses, thereby better guiding individualized clinical treatment strategies (Díaz et al., 2022). Furthermore, the combination of serine + isoleucine + betaine + PC(5:5/5:0) + LysoPE (18:2) was used to differentiate between acute ischemic stroke (AIS) and healthy controls, achieving an AUC of 0.971 in the test set (AUC = 0.988 in the training set). Therefore, combined analysis of LysoPE with other metabolites has shown potential in differentiating acute ischemic stroke from healthy controls (Ding et al., 2025). Despite these promising findings, integrating lipidomics into clinical practice faces several challenges. These include the lack of standardized protocols for sample preparation and data acquisition, difficulties in cross-laboratory reproducibility, the need for large-scale validation cohorts, and the complexity of distinguishing disease-specific signals from background variation. Furthermore, the dynamic range of LysoPE species and their sensitivity to pre-analytical factors (e.g., fasting status, sample processing time) require careful consideration in study design. Addressing these challenges through systematic multi-center studies will be essential for translating LysoPE biomarker discoveries into clinical applications.

### 10.2 Predictive value of LysoPE for treatment outcomes

In patients with multiple myeloma, significant changes in LysoPE levels reflect patient response to treatment. LysoPE (16:0) and LysoPE (18:2) may be associated with shorter progression-free survival (PFS), and LysoPE (16:0) is an independent risk marker for predicting early disease progression (Wei et al., 2022).

Moreover, LysoPE demonstrates application value in predicting drug-related adverse effects and guiding individualized treatment. In critically ill patients receiving tigecycline, changes in serum LysoPE levels can predict coagulation dysfunction, offering new insights for optimizing treatment regimens (Yang N. et al., 2025). In schizophrenia patients treated with the antipsychotic olanzapine, LysoPE levels were correlated with cognitive improvement, and baseline LysoPE (16:1) levels could serve as a predictive factor for cognitive enhancement following olanzapine monotherapy (Li et al., 2024).

## 11 Discussion

The findings consolidated in this review reveal that LysoPE is not merely a structural membrane component but a functionally versatile lysophospholipid involved in diverse physiological and pathological processes. Several key themes emerge from the literature. First, LysoPE exhibits disease- and context-dependent effects, ranging from pro-inflammatory to anti-inflammatory, and from pro-apoptotic to neuroprotective. Second, while lipidomics studies have consistently identified altered LysoPE levels across multiple diseases, much of the evidence remains correlational. Causal relationships have been established primarily through *in vitro* studies (e.g., MAPK activation in PC12 cells, chemotaxis in SK-OV3 cells), but *in vivo* loss-of-function experiments are largely lacking. Third, the receptor repertoire for LysoPE remains incompletely characterized, with evidence supporting LPAR1, GPR119, and potentially other unidentified GPCRs depending on cell type. This receptor promiscuity may explain the pleiotropic effects of LysoPE but also complicates therapeutic targeting.

Limitations of the current literature include small sample sizes in clinical studies, heterogeneity in LysoPE species analyzed, lack of standardized detection methods, and the predominance of descriptive lipidomics without mechanistic follow-up. Future studies should prioritize causal validation using genetic or pharmacological approaches, large-scale multi-center cohorts for biomarker qualification, and systematic investigation of LysoPE species-specific functions (Table 2).

**TABLE 2:** Critical appraisal of key studies on LysoPE functions.

Study (first author, year)	Pathway/Disease	Role of LysoPE	Supporting evidence	Correlation vs. causation	Weaknesses/Limitations
Yamamoto et al. (2021)	NAFLD	↓ LysoPE as diagnostic biomarker	LC-MS/MS serum analysis (n = 8–27)	Correlation	Small sample size, cross-sectional design
Nishina et al. (2006)	Neurotrophism	Activates MAPK pathway, promotes neurite outgrowth	PC12 cell culture, inhibitor studies (PD98059, U0126)	Causal (inhibitor evidence)	*In vitro* only, receptor not identified
Park et al. (2007)	Ovarian cancer	Promotes chemotactic migration and invasion	SK-OV3 cells, pertussis toxin (PTX) sensitivity	Causal (PTX inhibits effect)	Single cell line, *in vitro* only
Harm et al. (2023)	Cardiovascular disease	↑ LysoPE species associated with adverse events	Platelet lipidomics (n = 208), cox regression	Correlation	Retrospective, potential confounding factors
Tan et al. (2020)	Lysophospholipid biology	Comprehensive review of transport and signaling	Literature synthesis	Not applicable	Review article, no primary data

## 12 Conclusion

**FIGURE 4:**
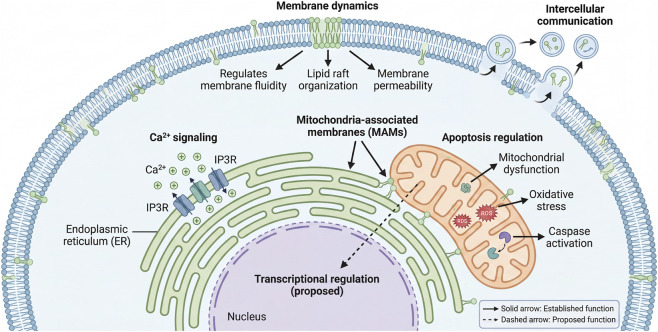
Subcellular distribution and functions of LysoPE. LysoPE exerts distinct functions depending on its subcellular localization. At the plasma membrane, it regulates membrane fluidity, lipid raft organization, and membrane permeability. In the endoplasmic reticulum (ER), LysoPE modulates Ca^2+^ release via IP3 receptors and contributes to mitochondria-associated membrane (MAM) function, which facilitates Ca^2+^ and lipid transfer between ER and mitochondria. At mitochondria, LysoPE influences apoptotic pathways, mitochondrial dysfunction, oxidative stress (ROS), and caspase activation. LysoPE is also present in extracellular vesicles, where it may participate in intercellular communication. In the nucleus, LysoPE may play a role in transcriptional regulation, but this function requires further validation. Solid arrows indicate established functions; dashed arrows indicate proposed roles requiring further validation.

In conclusion, LysoPE is a functionally versatile lysophospholipid that regulates membrane dynamics, signal transduction, and metabolic homeostasis. Its dysregulation is implicated in metabolic, neurodegenerative, cardiovascular, and neoplastic diseases, often in context-dependent and species-specific manners. While emerging evidence supports its promise as a candidate diagnostic and prognostic biomarker, significant knowledge gaps remain regarding its receptor repertoire, causal roles in disease pathogenesis, and clinical validation (Figure 4). Addressing these challenges through integrated multi-omics approaches, large-scale cohort studies, and mechanistic *in vivo* models will be essential to translate LysoPE biology into clinical applications.

## 13 Methods

This study employed a narrative review approach to comprehensively collate and analyze the pathophysiological roles of lysophosphatidylethanolamine (LysoPE) in diseases, along with its potential value as a biomarker. A literature search was conducted in databases including PubMed, Web of Science, Scopus, and Google Scholar, with no restrictions on the starting date to ensure the inclusion of both classic and cutting-edge studies. Search keywords consisted of Chinese and English terms and their combinations such as “lysophosphatidylethanolamine,” “LysoPE,” “LPE,” “biomarker,” “metabolic diseases,” “neurodegenerative diseases,” “signaling pathways,” and “lipidomics,” among others, and Boolean operators were used to construct compound search formulas. The literature screening process involved three steps: preliminary screening (based on titles and abstracts), full-text evaluation, and reference backtracking. The inclusion criteria covered experimental studies, clinical research, animal models, cell experiments, and mechanistic reviews, with a focus on research related to the structure, metabolism, functions, mechanisms, and clinical applications of LysoPE. Only peer-reviewed literature in Chinese and English was included. Conference abstracts, dissertations, commentaries, and studies that were either unavailable in full text or irrelevant in content were excluded. Key information such as research design, sample details, detection methods, main findings, and disease associations was extracted from each included study. Given the significant heterogeneity among the studies, this research adopted a narrative synthesis method for content integration and analysis, rather than performing a quantitative meta-analysis, aiming to systematically present the functional network and application prospects of LysoPE in different disease contexts.

## Glossary

Aβ: Amyloid-beta
ACC1: Acetyl-CoA carboxylase 1
AD: Alzheimer’s disease
AGHD: Adult growth hormone deficiency
AIS: Acute ischemic stroke
ATGL: Adipose triglyceride lipase
AUC: Area under the curve
BBB: Blood-brain barrier
BCPs: Black chokeberry polyphenols
BYHWD: Buyang Huanwu Decoction
CD36: Cluster of differentiation 36
CH: Cardiac hypertrophy
CIA: Collagen-induced arthritis
COX-2: Cyclooxygenase-2
CPT1α: Carnitine palmitoyltransferase 1 alpha
CRC: Colorectal cancer
DHA: Docosahexaenoic acid
EGFR: Epidermal growth factor receptor
EMT: Epithelial-mesenchymal transition
EPT1: Ethanolaminephosphotransferase 1
ERK1/2: Extracellular signal-regulated kinases 1/2
EUE: Eucommia ulmoides extract
GPCRs: G-protein-coupled receptors
GPR119: G protein-coupled receptor 119
HGyp: Heat-processed Gynostemma pentaphyllum
HIF-1α: Hypoxia-inducible factor 1-alpha
HIV: Human immunodeficiency virus
HOMA-IR: Homeostatic model assessment for insulin resistance
iGCTs: Intracranial germ cell tumors
IFN-γ: Interferon-gamma
IL-1β: Interleukin-1 beta
IL-6: Interleukin-6
iNOS: Inducible nitric oxide synthase
IR: Ischemia-reperfusion
LCAT: Lecithin-cholesterol acyltransferase
LC-MS/MS: Liquid chromatography-tandem mass spectrometry
LPAR1/LPA1: Lysophosphatidic acid receptor 1
**Lp-PLA**_**2**_: Lipoprotein-associated phospholipase A_2_
LysoPC: Lysophosphatidylcholine
LysoPE: Lysophosphatidylethanolamine
MAMs: Mitochondria-associated endoplasmic reticulum membranes
MAPK: Mitogen-activated protein kinase
Mfsd2a: Major facilitator superfamily domain containing 2A
mTOR: Mechanistic target of rapamycin
NACT: Neoadjuvant chemotherapy
NAFLD: Non-alcoholic fatty liver disease
NASH: Non-alcoholic steatohepatitis
NGF: Nerve growth factor
NF-M: Neurofilament-medium
OxPLs: Oxidized phospholipids
PAF-AH: Platelet-activating factor acetylhydrolase
PC: Phosphatidylcholine
PE: Phosphatidylethanolamine
PFBS: Perfluorobutane sulfonate
PFS: Progression-free survival
PI3K/AKT: Phosphoinositide 3-kinase/Protein kinase B
**PLA**_**2**_: Phospholipase A_2_
PLD: Phospholipase D
PPARα: Peroxisome proliferator-activated receptor alpha
PPARγ: Peroxisome proliferator-activated receptor gamma
RA: Rheumatoid arthritis
ROC: Receiver operating characteristic
ROS: Reactive oxygen species
Sal B: Salvianolic acid B
SCD1: Stearoyl-CoA desaturase 1
SDD: Subretinal drusenoid deposits
siRNA: Small interfering RNA
SLE: Systemic lupus erythematosus
**sPLA**_**2**_: Secretory phospholipase A_2_
SREBP1: Sterol regulatory element-binding protein 1
STAT3: Signal transducer and activator of transcription 3
TGF-β: Transforming growth factor beta
TIC: Trauma-induced coagulopathy
TNF-α: Tumor necrosis factor-alpha
VEGF: Vascular endothelial growth factor
